# Supplementary material for: Hybrid Models and Biological Model Reduction with PyDSTool
Source: PLoS Comput Biol. 2012 Aug 9;8(8):e1002628. doi: 10.1371/journal.pcbi.1002628 (PMC3415397; doi:10.1371/journal.pcbi.1002628)
Supplement: Text S4 — Complete source code for the PyDSTool package (version 0.88.120504). Includes API documentation and help files linking to web pages. This file is identical to the current public release on Sourceforge.net. (ZIP) [file pcbi.1002628.s004.zip › PyDSTool/html/PyDSTool.PyCont.Continuation.EquilibriumCurve-class.html]

xml version="1.0" encoding="ascii"?


PyDSTool.PyCont.Continuation.EquilibriumCurve


| Home | Trees | Indices | Help | PyDSTool |
| --- | --- | --- | --- | --- |

Package PyDSTool ::
Package PyCont ::
Module Continuation ::
Class EquilibriumCurve

[hide private]

[frames] | no frames]

# Class EquilibriumCurve

source code

```
  object --+    
           |    
Continuation --+
               |
              EquilibriumCurve
```

---

```
Child of Continuation class that represents curves of
equilibrium points.

System:

        h(x,a) = f(x,a) = 0

    The continuation variables are (x,a) with

        x = State variables
        a = Free parameters

Detection of points: LP, H, BP
```


|  |  |
| --- | --- |
| Instance Methods | [hide private] |

|  |  |
| --- | --- |
| \_\_init\_\_(self, model, gen, automod, plot, args=None)  x.\_\_init\_\_(...) initializes x; see x.\_\_class\_\_.\_\_doc\_\_ for signature | source code |

|  |  |
| --- | --- |
| reset(self, args=None)  Resets curve by setting default parameters and deleting solution curve. | source code |

|  |  |
| --- | --- |
| update(self, args)  Update parameters for EquilibriumCurve. | source code |

|  |  |
| --- | --- |
| \_createTestFuncs(self)  Creates processors and test functions for EquilibriumCurve class. | source code |

**Inherited from `Continuation`**:
`__copy__`,
`__deepcopy__`,
`__repr__`,
`__str__`,
`backward`,
`cleanLabels`,
`computeEigen`,
`display`,
`exportGeomview`,
`forward`,
`getSpecialPoint`,
`info`,
`testdomain`,
`testdomaingrid`,
`testdomaintangrid`

**Inherited from `Continuation`** (private):
`_Keller`,
`_MoorePenrose`,
`_Natural`,
`_checkForBifPoints`,
`_compute`,
`_curveToPointset`,
`_preTestFunc`,
`_savePointInfo`,
`_system`,
`_systemjac`,
`_systemjac_withpars`,
`_systemjacuser`,
`_systemuser`

**Inherited from `object`**:
`__delattr__`,
`__getattribute__`,
`__hash__`,
`__new__`,
`__reduce__`,
`__reduce_ex__`,
`__setattr__`


|  |  |
| --- | --- |
| Properties | [hide private] |

**Inherited from `object`**:
`__class__`


|  |  |
| --- | --- |
| Method Details | [hide private] |

|  |  |
| --- | --- |
| \_\_init\_\_(self, model, gen, automod, plot, args=None)  *(Constructor)* | source code |

x.\_\_init\_\_(...) initializes x; see x.\_\_class\_\_.\_\_doc\_\_ for
signature

Overrides: object.\_\_init\_\_: *(inherited documentation)*

|  |  |
| --- | --- |
| reset(self, args=None) | source code |

Resets curve by setting default parameters and deleting solution
curve.

Overrides: Continuation.reset

|  |  |
| --- | --- |
| update(self, args) | source code |

Update parameters for EquilibriumCurve.

Overrides: Continuation.update

|  |  |
| --- | --- |
| \_createTestFuncs(self) | source code |

```
Creates processors and test functions for EquilibriumCurve
class.

Note:  In the following list, processors are in
PyCont.Bifpoint and test functions are in PyCont.TestFunc.

Point type (Processor): Test function(s)
----------------------------------------

LP (FoldPoint): Fold_Tan
                Fold_Det
                Fold_Bor
H (HopfPoint): Hopf_Det
               Hopf_Bor
```

Overrides: Continuation.\_createTestFuncs

  


| Home | Trees | Indices | Help | PyDSTool |
| --- | --- | --- | --- | --- |

|  |  |
| --- | --- |
| Generated by Epydoc 3.0.1 on Fri May 4 15:24:07 2012 | http://epydoc.sourceforge.net |
